# Unleashing the potential of fNIRS with machine learning: classification of fine anatomical movements to empower future brain-computer interface

**DOI:** 10.3389/fnhum.2024.1354143

**Published:** 2024-02-16

**Authors:** Haroon Khan, Rabindra Khadka, Malik Shahid Sultan, Anis Yazidi, Hernando Ombao, Peyman Mirtaheri

**Affiliations:** ^1^Department of Mechanical, Electronics and Chemical Engineering, OsloMet - Oslo Metropolitan University, Oslo, Norway; ^2^Department of Information Technology, Oslomet - Oslo Metropolitan University, Oslo, Norway; ^3^Department of Computer, Electrical and Mathematical Science and Engineering, King Abdullah University of Science and Technology (KAUST), Thuwal, Saudi Arabia

**Keywords:** functional near-infrared spectroscopy (fNIRS), deep learning, individual finger movements, classification, brain computer-interface (BCI)

## Abstract

In this study, we explore the potential of using functional near-infrared spectroscopy (fNIRS) signals in conjunction with modern machine-learning techniques to classify specific anatomical movements to increase the number of control commands for a possible fNIRS-based brain-computer interface (BCI) applications. The study focuses on novel individual finger-tapping, a well-known task in fNIRS and fMRI studies, but limited to left/right or few fingers. Twenty-four right-handed participants performed the individual finger-tapping task. Data were recorded by using sixteen sources and detectors placed over the motor cortex according to the 10-10 international system. The event's average oxygenated Δ HbO and deoxygenated Δ HbR hemoglobin data were utilized as features to assess the performance of diverse machine learning (ML) models in a challenging multi-class classification setting. These methods include LDA, QDA, MNLR, XGBoost, and RF. A new DL-based model named “Hemo-Net” has been proposed which consists of multiple parallel convolution layers with different filters to extract the features. This paper aims to explore the efficacy of using fNRIS along with ML/DL methods in a multi-class classification task. Complex models like RF, XGBoost, and Hemo-Net produce relatively higher test set accuracy when compared to LDA, MNLR, and QDA. Hemo-Net has depicted a superior performance achieving the highest test set accuracy of 76%, however, in this work, we do not aim at improving the accuracies of models rather we are interested in exploring if fNIRS has the neural signatures to help modern ML/DL methods in multi-class classification which can lead to applications like brain-computer interfaces. Multi-class classification of fine anatomical movements, such as individual finger movements, is difficult to classify with fNIRS data. Traditional ML models like MNLR and LDA show inferior performance compared to the ensemble-based methods of RF and XGBoost. DL-based method Hemo-Net outperforms all methods evaluated in this study and demonstrates a promising future for fNIRS-based BCI applications.

## 1 Introduction

fNIRS (functional near-infrared spectroscopy) is a non-invasive neuroimaging technique that uses near-infrared light to measure changes in oxygenated (Δ HbO) and deoxygenated hemoglobin (Δ HbR) in the brain (Ferrari and Quaresima, 2012; Wilcox and Biondi, 2015). Neural activity in a brain region is associated with blood flow changes due to neurovascular responses; fNIRS measures the brain activation by using near-infrared light (optical window of wavelength: 650 − 1100; nm) (Strangman et al., 2003; Sato et al., 2004). The change in the optical densities is then converted to hemoglobin concentration changes using Modified Beer-lambert Law (MBBL) (Wilcox and Biondi, 2015). fNIRS is becoming popular in brain-computer interface (BCI) research because it is non-invasive, portable, and relatively low-cost compared to other neuroimaging techniques such as functional magnetic resonance imaging (fMRI) (Naseer and Hong, 2015; Khan et al., 2021a). In early 2000, it was potentially complemented that the new developments in fMRI equipment, preprocessing algorithms, and robust statistical approaches will make it suitable for BCI applications (Sitaram et al., 2007). However, with its limitation of temporal resolution, restricted movements, constraint due to strong magnetic field, and calibration issues, fNIRS is becoming more popular for BCI applications compared to fMRI. The fNIRS measurements of concentration changes yield a similar signal as the blood oxygen level-dependent (BOLD) response acquired through fMRI with additional information about Δ HbR (Strangman et al., 2002; Gagnon et al., 2012). Additionally, it can measure changes in brain activity at a relatively high temporal resolution, which is important for real-time BCI applications.

Among the main challenges in fNIRS-based BCI include a reduction in the response time, an increase in the number of control commands, and improving the classification accuracy of the system (Hong and Khan, 2017). To achieve these goals and utilize the advantage of different brain imaging modalities for BCI applications, a new sub-field emerged within BCI called hybrid BCI (hBCI). In hybrid BCI, at least two brain signal modalities are combined with one another (Pfurtscheller et al., 2010; Hong et al., 2018). Different modalities, such as EEG-fMRI and EEG-fNIRS, have been merged to enhance the BCI system. Considerable technical and analytical developments have made valuable scientific contributions to the field of BCI, but due to some series of technical challenges such as fMRI magnetic field interface with EEG signals, portability, and other safety concerns due to strong magnetic field made it limited (Warbrick, 2022). On the other hand, fNIRS technology can easily be combined with other modalities, such as electroencephalogram (EEG) and Electromyography (EMG), to provide a better picture of brain activity. There is evidence that combined EEG, and fNIRS-based BCI systems perform better than individual EEG and fNIRS-based BCI systems. although it should be noted that it is still necessary to conduct further research to understand the physiological interactions of EEG and fNIRS in detail. Hybrid EEG-fNIRS setups are fraught with problems based on the differences in measurement methods and the ground differences of the nature of these biosignals in two different domains (temporal and spatial) that must be analyzed simultaneously (Ahn and Jun, 2017; Khan et al., 2021a). The focus of our study is to individually explore the potential of information in fNIRS signals to increase the number of control commands. However, our future work will explore the individual finger-tapping task with a hybrid EEG-fNIRS study.

In this study, we investigate the possibility of utilizing fNIRS signals from individual channels with advanced machine-learning techniques to classify specific anatomical movements instead of relying on hybrid modalities to increase the number of control commands for BCI applications. To achieve this, we focus on a well-known task in fNIRS and fMRI studies called finger tapping. To make the task more complex, we focus on the fine anatomical movements, such as individual finger movements, which to the author's knowledge, have never been explored with fNIRS. fNIRS is more feasible to use in a naturalistic setting where the participant can move around compared with an fMRI environment. The reason is very apparent that fNIRS has very low spatial resolution compared to fMRI. It is worth noting that each body part has a distinct area in the primary motor cortex dedicated to it. However, due to its lower spatial resolution than fMRI, accurately classifying individual finger movements using fNIRS alone presents challenges. fNIRS as a single modality has the advantage of supplying additional information about Δ*HbR*, which could possibly be used to estimate the metabolic rate (Boas et al., 2003). Nevertheless, we hypothesize that the motor cortex signals contain valuable information that can be leveraged for enhancing control commands through modern machine learning algorithms.

Till 2017, deep learning methods did not show any significant improvements compared to state-of-the-art techniques used for bio-signals classification in BCI (Lotte et al., 2018). However, recent research shows its future potential due to its ability to simultaneously learn useful features and classifiers from raw data. Based on the potential of the deep learning model, this research hypothesizes that fNIRS signal features can be used to distinguish even finite movements, such as individual finger tapping. This will be useful for various future applications in the field of fNIRS-based BCI. The increase in classification accuracy and control command generation is significant in BCI because they directly impact the usability and effectiveness of the system. Classification accuracy of the BCI system refers to the ability to correctly identify and interpret the user's intent from their brain signals (Lotte et al., 2007). The finger tapping task is a well-understood and relatively simple motor task with specific brain activation patterns used in the BCI experiment (Middendorf et al., 2000). But even for such tasks, detecting delicate anatomical structures, such as recognizing individual finger movements in BCI applications, is a complex and ongoing area of research. So far, continuous individual finger movements decoding and control in BCI are achieved using EMG signals, but it cannot be achieved in the case of muscle paralysis. On the other hand, invasive brain signal modalities, Electrocorticography (ECoG)-based BCI, shows promising results in distinguishing between individual finger movements due to its good spatio-spectral features, such as discriminating between ipsilateral or contralateral along with thumb or index finger movements (Zanos et al., 2008). In another study, using ECoG arrays, the intention for individual finger movements was classified using LDA with an overall accuracy of 67% for people with tetraplegia to use the BCI system accuracy (Jorge et al., 2020). However, these invasive methods are not feasible for BCI applications. To develop a deeper understanding of the motor control system during the individual finger-tapping exercise and to classify the specific finger movements, we will explore state-of-the-art data analytic tools. Machine Learning (ML) is characterized by learning hidden patterns from the data. In supervised ML the labels for the target variable are known in the data, and a function or model is learned to map the input to the output space. The targets can be continuous in the case of regression and discrete in classification. Similarly, the input variables can be pixels of an image, a portion of a time series, coefficients of statistical models, statistical summaries of data, Fourier and Wavelet transformations, etc. Many traditional statistical techniques and machine learning algorithms such as Linear Regression, Logistic Regression, K-Nearest Neighbors, and other available methods, rely on pre-processed features for learning the classification and regression functions. In a recent study using the modern machine learning approach, an improved and fast decoding of all five fingers along with resting state (six class) with a classification accuracy of 77% was achieved using ECoG (Yao et al., 2022). However, ECoG is limited due to its invasive nature. Therefore, inspired by ECoG, researchers attempted to classify non-invasively using EEG to decode individual finger movements. Liao et al. (2014) decode individual finger movements from one hand with an average accuracy of 77.11% using binary classification and support vector machine (SVM) as a classifier to classify between the pair of fingers using spectral changes in EEG data. In a recent study using a high-density EEG electrode setup, the average classification (SVM as a classifier) accuracy achieved using pairwise finger was 64.8%. Most other EEG studies demonstrate decoding of multiple finger movements to enhance the control command generation for the BCI system instead of single or contralateral finger movements (Gannouni et al., 2020). All these findings motivate the investigation of individual finger movements with fNIRS that can be utilized in various fNIRS-based BCI applications such as prosthetic arm development and rehabilitation.

The fMRI has a comparable high spatial resolution to fNIRS demonstrating reasonable classification accuracies for the classification of related tasks. In a study using real-time whole-brain imaging the right and left index finger movements were classified with an accuracy of 80% (LaConte et al., 2007). Decoding individual finger movements from the right hand using single-trial fMRI data was processed in one study using multivariate pattern classification analysis approaches (Shen et al., 2014). The average accuracy of 63% (best trail 84.7%) was achieved to classify between five fingers. Another study using fMRI data demonstrated that hyperalignment provides better between-subject classification accuracy of 88.8% than conventional anatomical alignment 46% using the four (index to little) finger presses movements (Kilmarx et al., 2021). Due to fNIRS's relatively lower spatial resolution, empirical data on how fine anatomical movements can be decoded from hemodynamic responses have yet to be investigated. In this research, we hypothesize that the fNIRS signal, with its rich information, could be used to distinguish delicate anatomical structures with modern classification algorithms to help enhance control commands for BCI systems, for example, the control of prosthetic hands and finger movements. The current fNIRS-based BCI research is limited to tapping one or more fingers, single or both hand-tapping, right and left finger-tapping, or hand-tapping. Significantly higher classification accuracy has been achieved using two classes (98.7 ± 1%, left vs. right finger tapping) and three classes (98.7 ± 6.9%, left versus right finger tapping vs. rest condition) problems using vector-based phase analysis (Nazeer et al., 2020). Using statistical features along with Δ*HbO* and Δ*HbR* data is also one of the common approaches to enhance classification accuracy even for motor imaginary (MI) tasks (Shin and Jeong, 2014). In a single-trail MI study, a finger tapping task was used to discriminate between thumb tapping vs. complex sequential tapping task with an accuracy of 81% (Holper and Wolf, 2011). In our pilot study on the collected dataset, we classified the individual finger movements against the baseline with classical machine learning algorithms such as SVM, random forests (RF), AdaBoost, (SVM), random forests (RF), decision trees (DT), AdaBoost, quadratic discriminant analysis (QDA), artificial neural networks (ANN), k-nearest neighbors (kNN), Artificial neural networks (ANN), and k-nearest neighbors (kNN). The average classification accuracies achieved were 75 ± 4%, 75 ± 5%, and 77 ± 6% using kNN, RF, and XGBoost, respectively (Khan et al., 2021b). The current study performed a classification of in-between finger movements, which is more difficult than finger movements with baseline or resting states. Comparing fNIRS with fMRI data, the misclassification rate is comparable for hand or finger movement tasks. However, as noted, fNIRS is a more promising modality because of its portability, relatively low cost, and its amenability to more naturalistic settings.

In this paper, we explored machine and deep learning (DL) models to classify fine anatomical movement (individual finger tapping) using fNIRS data. There are two primary difficulties: firstly, the classification of such minute movements using fNIRS, and secondly, the classification is a multi-class problem (classifying five fingers and the resting state). DL is inspired by information processing in the human brain; these models can learn complex and non-linear functions. It can potentially learn complex non-linear interactions between brain regions; and associations between the fNIRS signal and the motor movement. Moreover, feature extraction is usually not required for DL models as the feature are extracted hierarchically in the hidden layers, where the initial layers learn the low-level features and the deep layers learn more complex high-level features corresponding to the task and loss function (Zeiler and Fergus, 2014). Therefore, these models learn the best representations from the data in the hidden layers. The study will be a foundation for classifying fine anatomical movement using fNIRS-based BCI. The paper is structured to describe the methodology in Section 2, results and discussion in Section 3, limitation of the current work in Section 4, and conclusion Section in 5.

## 2 Materials and methods

In this section, we describe the methods used for data collection, experimental design, and data analysis.

### 2.1 Participant recruitment and training

The experiment involved 24 healthy right-handed participants, 18 males (M = 30.44 ± 3.03 in years; range: 24 to 34 years old) and six females (F = 29.17 ± 3.06 in years; range: 24 to 34 years old). It was required that participants write with their right hands with no neurological disorders or limitations in hand or finger motor abilities to meet the inclusion criteria. All the participants performed the finger tapping with finger from right hand only. The experiment was performed in a relatively controlled environment such as a quiet room to reduce any attentional bias, and a black shower cap to reduce the background noise from lamps and computer screens. A visual presentation in the textual format of resting and task (finger name to tap) was displayed on the computer monitor. Experiments were preceded by practice sessions in which participants were informed of the protocol and procedures. Medium-to-fast finger tapping was performed without any specific frequency. Experiments were repeated for each participant based on their comfort and convenience. The experiment followed the declaration of Helsinki. Research Ethics Committee (REK No. 322236) no objection letter was obtained for experimental work. According to the Norwegian Center for Research Data AS (NSD Ref. No. 647457), informed consent for voluntary participation was given by all the participants before the experiment. Further details can be found in Khan et al. (2021b).

### 2.2 Instrumentation, experimental paradigm, and montage

**Figure 1:**
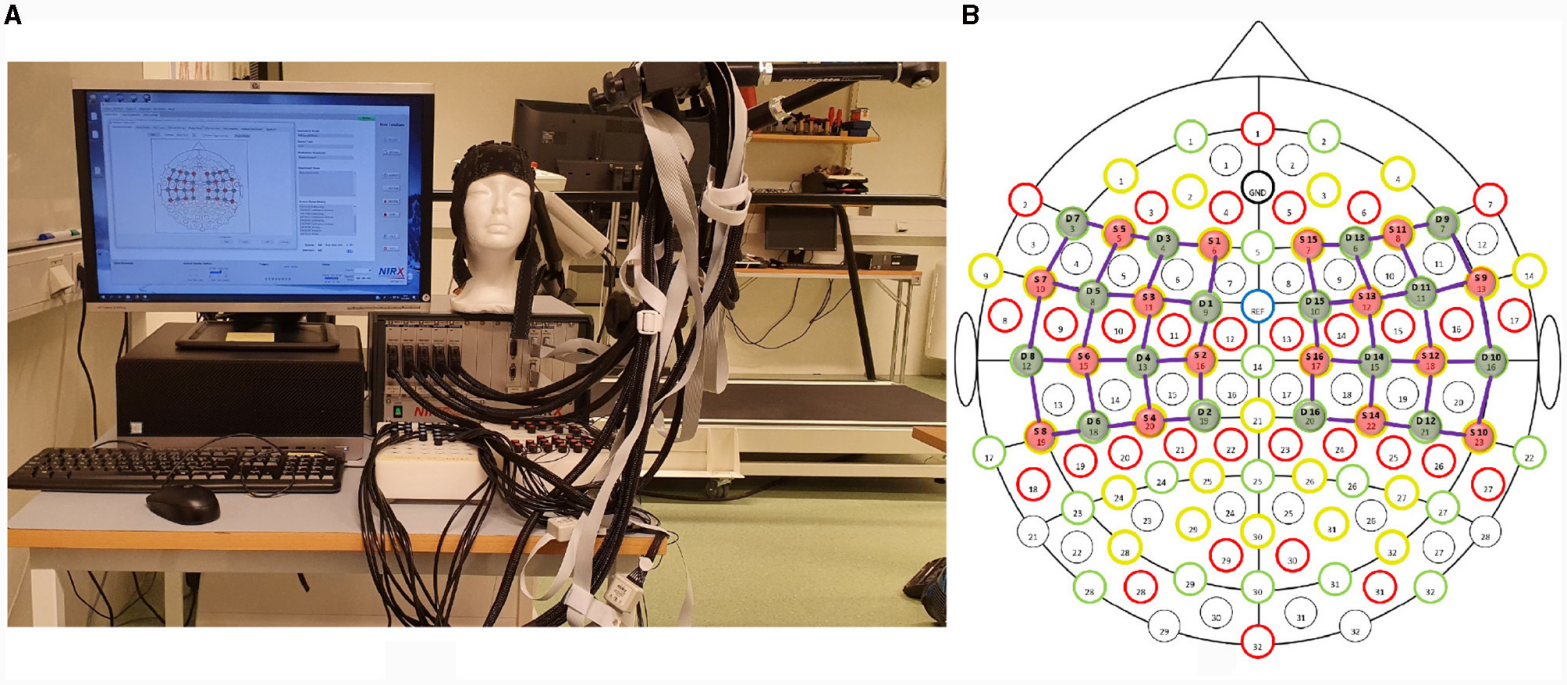
**(A)** Experimental setup demonstrating fNIRS NIRScout (NIRx Medical Technology GmbH, Germany) **(B)** Sixteen sources and detectors each were placed over the motor cortex according to the 10 − 10 international system.

**Figure 2:**
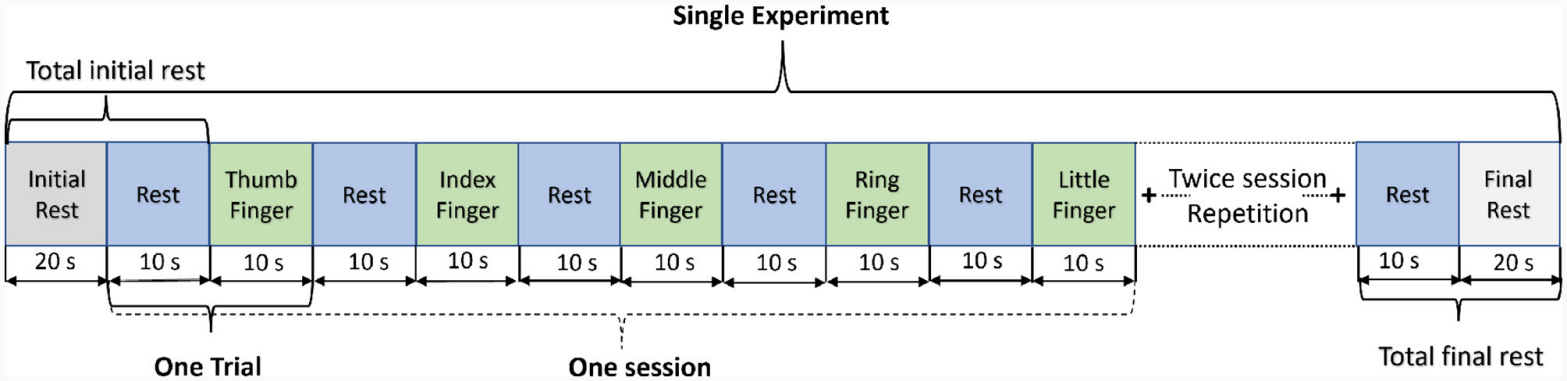
Experimental paradigm: a single run of the experiment includes three repetitive sessions of each finger-tapping.

A continuous-wave (λ_1_ = 760*nm*, λ_2_ = 850*nm*) optical tomography machine NIRScout (NIRx Medizintechnik GmbH, Germany) was used to acquire brain data with a sampling rate of 3.9063 Hz as shown in Figure 1A. The block design consists of blocks of rest and task (thumb, index, middle, ring, and little finger-tapping) of the right hand, as shown in Figure 2. A baseline rest of 30 sec and a tapping duration of 10 sec was performed to achieve a robust hemodynamic response in response to finger-tapping activity (Khan et al., 2020). The single experimental paradigm consisted of three sessions of each finger-tapping trial. The total length of the experiment was 350 sec. The single trial included 10 sec of rest followed by 10 sec of the task. Before placing the fNIRS cap on the participant's heads, cranial landmarks (inion and nasion) were marked to locate *C*_*z*_. The emitter and detector were placed according to the 10–10 International electrode positioning layout. The distance between the source and the detector was kept at a minimum of 3 cm using optode holders. Sixteen emitters and detectors were positioned over the motor cortex according to standard *motor*_16*x*16_ shown in Figure 1B. The source detectors are assumed to cover the part of the frontal lobe, frontal-central sulcus lobe, central sulcus lobe, part of the central-parietal lobe, and temporal-parietal lobe.

### 2.3 Signal prepossessing

**Figure 3:**
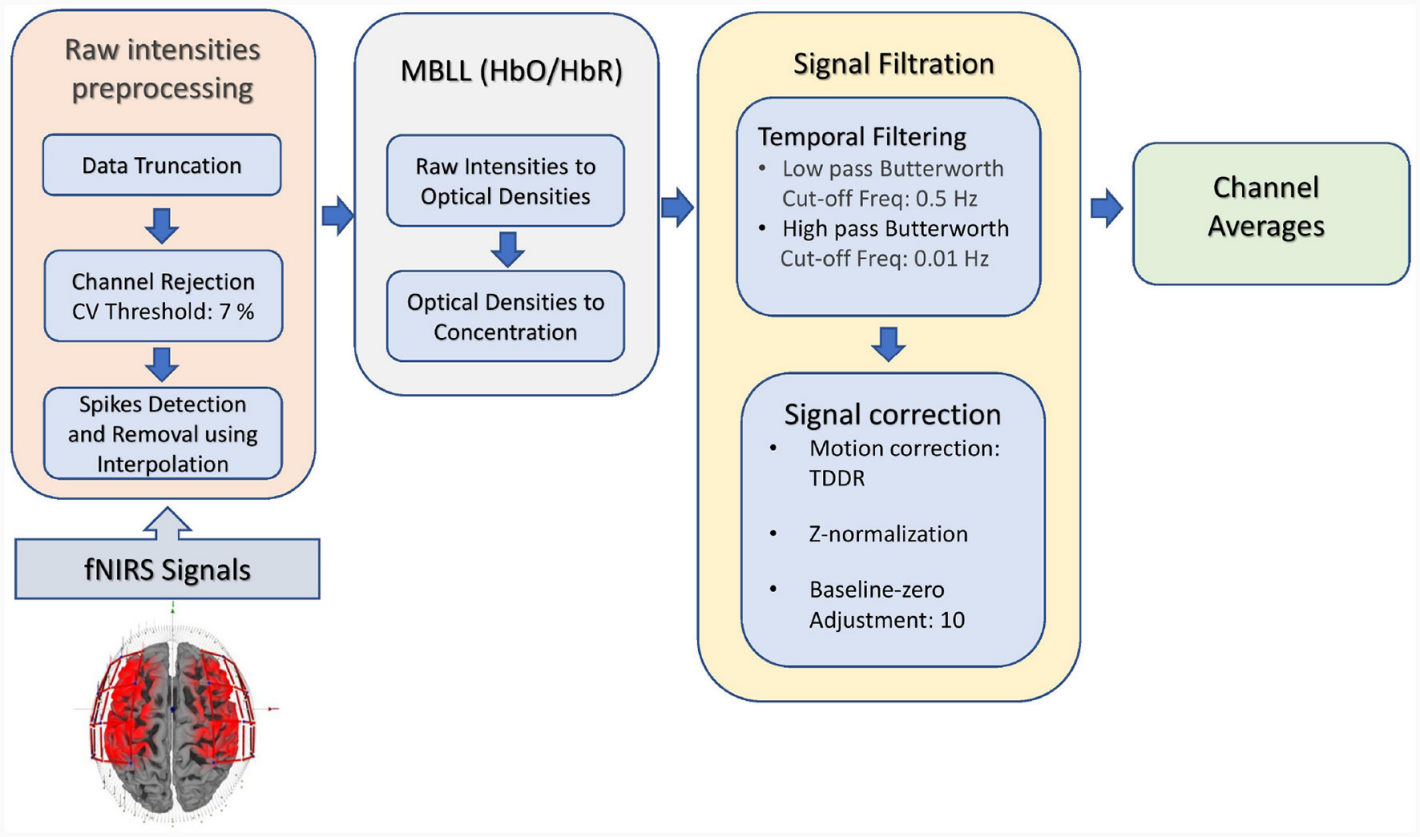
Data processing steps followed before application of DL model.

fNIRS data were processed using the pipeline as shown in Figure 3. The data processing included data truncation (removal of data points before and after the first and last stimuli appeared, respectively), spike removal (using the interpolation method), and channel rejection based on the criteria of coefficient of variation (CV) of 7.5%. The Modified Beer-Lambert Law (MBBL) converted optical densities into hemoglobin concentration changes. The spontaneous contamination of physiological and non-physiological noise was removed using the Butterworth filter (low-pass frequency: 0.01 Hz, high-pass frequency: 0.5 Hz). Data were then cleaned for motion noises by applying temporal-derivative distribution repair (Fishburn et al., 2019). Signal correction such as z-normalization and baseline-zero adjustments (value = 10) was performed.

### 2.4 Classification

#### 2.4.1 Event-related averages

After the signal processing step mentioned in Section 2.3 the filtered event averages from each run corresponding to each finger-tapping task were calculated. The task was averaged into epochs from 5 sec before stimulus onset to 15 sec (including 10 sec task duration and 5 sec of post-task) ensuring no conflict with the onset of the next stimulus presentation, which may occur as early as 20 sec after the previous stimulus onset. The channel-specific event averages for Δ*HbO*, Δ*HbR*, and Δ*HbT* are considered features of the machine learning model presented in the upcoming section.

#### 2.4.2 Classifier

Time series classification (TSC) deals with classifying time series as belonging to specific classes. Given dataset *D* = (*X*_*i*_, *Y*_*i*_), the goal in TSC is to learn a mapping function (f: X → Y), this mapping function is called a classifier. Machine learning was used to learn the classification function to predict the class probabilities given the feature vector *X*_*i*_. In the case of multivariate time series, a single training example $X_{i}\in {\mathbb{R}}^{m\times t}$, where *m* is the total number of time series, and *t* is the length of each time series in the data. The problem of finger-taping can be modeled as a multi-class classification problem, in the present case *X*_*i*_ is a 1 × 77-dimensional vector representing the oxygenation level at discrete time steps of an individual channel. Mathematically, the classification problem can be written as


(1) $$\begin{array}{l} P\left(Y=y_{i}|X=x_{i}\right)=f\left(X=x_{i},w\right) \end{array}$$

where *Y*, is the target vector containing the class labels for the 5-finger taping respectively. *X* is the feature vector, and *f*(*X*) is the classification function which is learned from the data, *w* are the parameters of the model that need to be learned to build this classifier, *x*_*i*_ is the event averages from one of the channels and *y*_*i*_ is the label for the *i*-th training example. The classification function can be learned using parametric models like a deep learning (DL) model, parameterized by the weights *w* of the neural network, or non-parametric models like KNN. The length of the time series (event-related averages) considered for each channel is 77-time points corresponding to the event average data of 20 sec of data for each tapping task as mentioned in Section 2.4.1. We are interested in the class conditional probability of a specific finger tapping given one of the channel's time series data.

#### 2.4.3 Data preparation

Deep Learning (DL) also known as representation learning is end-to-end learning. The traditional ML models require feature extraction and engineering, which are generally not required for DL models, as the features are learned in hidden layers. The DL models learn by forward and back-propagation of errors, in which gradients are calculated to update the model's weights until a convergent solution is obtained for minimizing the loss function. Scaling of the input or feature vector is required for the DL models for faster convergence when using gradient-based methods and comparable input features. For this reason, we scaled the input feature vector using standard scaling.

#### 2.4.4 Model evaluation

The classifier's evaluation metrics are mainly accuracy, precision, and recall. In a classification problem, accuracy is defined as the ratio of correctly classified samples to the total number of samples in the data. Define *Y*_*p, i*_ and *Y*_*t, i*_ to be the predicted label and true label for the *i*-th sample and *N* be the total number of examples, then accuracy is defined mathematically as


(2) $$\begin{array}{l} Accuracy=\frac{\sum I\left(Y_{p,i}==Y_{t,i}\right)}{N} \end{array}$$

where “I” is an indicator function. Precision is defined as the ratio of the true positives in the data to the total number of samples predicted as positives by the classifier. For a class k, let *T*_*p*_ be the total number of samples belonging to class k that the model has accurately predicted, *T*_*n*_ be the total number of samples correctly predicted as belonging to other classes, *F*_*p*_ be the total number of instances of wrong predictions made by the model as belonging to class k, and *F*_*n*_ be the total number of instances of wrong predictions made by the model as belonging to other classes. Then precision is defined to be


(3) $$\begin{array}{l} Precision=\frac{T_{p}}{T_{p}+F_{p}} \end{array}$$

The recall is also known as sensitivity and is defined as the ratio of the true positives to the sum of true positives and false negatives, recall is defined as follows


(4) $$\begin{array}{l} Recall=\frac{T_{p}}{T_{p}+F_{n}}. \end{array}$$

### 2.5 Machine learning classifiers

This section gives the details of various ML-based multi-class classifiers, built for the classification of individual finger tapping. Our analysis compares the performance of Random Forest (RF), XGBoost, Linear Discriminant Analysis (LDA), Quadratic Discriminant Analysis (QDA), and Multinomial Logistic Regression (MNLR).

#### 2.5.1 Linear discriminant analysis

Linear Discriminant Analysis (LDA) is a supervised machine learning algorithm for classification (Fisher, 1936; Johnson et al., 2002). In LDA, classification is viewed as a problem of dimension reduction. It finds the linear boundary that separates the classes by learning the discriminant functions by projecting data in a lower dimensional space. The LDA finds the best projections using discriminant functions to maximize the separation between classes (the means of the projected vectors) and minimize the variance within a class. Let *X* = *x*_1_, *x*_2_, …, *x*_*N*_ be the data where $x_{i}\in R^{d}$, and *Y* = *y*_1_, *y*_2_, …, *y*_*N*_ be the class labels for K classes respectively. Let μ_*k*_ be the class-specific mean, and μ be the overall mean of the data.


(5) $${\hat{S}}_{W}=\sum_{k=1}^{K}\sum_{x\in k}(x-\mu_{k}){\left(x-\mu_{k}\right)}^{T}$$

where *Ŝ*_*W*_ represents the variance within each class also called within class scatter matrix. The between-class variance or scatter matrix, which has to be maximized is given by the following relation


(6) $${\hat{S}}_{B}=\sum_{k=1}^{K}n_{k}(\mu_{k}-\mu ){\left(\mu_{k}-\mu \right)}^{T}$$

where *Ŝ*_*B*_ is the between class variance matrix, and *n*_*k*_ is the number of examples in class *k*, μ is the overall mean vector. The projection matrix *W* can be obtained by solving the generalized eigenvalue problem given in Equation 7, by selecting the largest *M* λ_*i*_ eigenvalues and corresponding *v*_*i*_ eigenvectors.


(7) $$({\hat{S}}_{W}^{-1}{\hat{S}}_{B})v=\lambda v$$

We applied 5-fold cross-validation and values of solver [*svd, lsqr, eigen*] and shrinkage [*auto, none*] hyper-parameters were tuned using grid search.

#### 2.5.2 Quadratic discriminant analysis

The Quadratic Discriminant Analysis (QDA) is a supervised learning classification algorithm, an extension of LDA that relaxes the equal variance assumption across all classes (Hastie et al., 2009). For each class, the QDA computes the class conditional densities separately and makes predictions using the Bayes Rule. In QDA, the class conditional densities are modeled using multi-variate Gaussian distributions. Let π_*k*_ be the prior probability for a training sample *X*_*i*_ belonging to class *k*. These prior probabilities for each class can be estimated from the data equivalent to the proportion of the data containing the sample belonging to the class *k*. Let μ_*k*_ represent the class-specific mean, and Σ_*k*_ be the class-specific covariance matrix. The posterior probability for a sample *X*_*i*_ as belonging to class *k* can be computed using Bayes Rule as follows:


(8) $$P(Y_{i}=k|X=X_{i})=\frac{\pi_{k}\cdot f_{k}(X_{i})}{\sum_{l=1}^{K}\left(\pi_{l}\cdot f_{l}(X_{i})\right)}$$

where *f*_*k*_ is a multivariate Gaussian density function corresponding to class k, defined to be


(9) $$\begin{array}{l} f_{k}\left(X_{i}\right)=\frac{1}{{\left(2\pi \right)}^{\frac{z}{2}}\cdot |\Sigma_{k}{|}^{\frac{1}{2}}}\cdot \exp\left(-\frac{1}{2}{\left(X_{i}-\mu_{k}\right)}^{T}\cdot \Sigma_{k}^{-1}\cdot \left(X_{i}-\mu_{k}\right)\right) \end{array}$$

where *z* indicates the dimension of the vector *X*_*i*_, each *X*_*i*_, μ_*k*_ ∈ *R*^*z*^. The 5-fold cross-validation was used during training, for tuning hyper-parameter reg param [0.0, 0.1, 0.5, 1.0] which controls the regularization on the covariance estimates was tuned using grid search.

#### 2.5.3 XGBoost

Extreme Gradient Boosting (XGBoost) is an ensemble learning model widely used for supervised machine learning, i.e., classification and regression problems (Chen and Guestrin, 2016). The goal of XGBoost is to minimize the cross-entropy loss, which can be stated as follows


(10) $$\begin{array}{l} L\left(Y_{t},Y_{p}\right)=-\overset{N}{\underset{i=1}{\sum }}\overset{K}{\underset{k=1}{\sum }}\left[y_{it}\log y_{ip}\right] \end{array}$$

where *y*_*i*_*p* is the predicted class probability or the predicted label for the instance *i*. The vector *y*_*it*_ for true class labels is one hot encoded with a dimension equal to *K* the number of classes. DT is used as base learners in the XGBoost algorithm, and the splits are performed based on the reduction in the loss. The 5-fold cross-validation was used during training, hyper-parameters including the number of estimators [50, 100], maximum depth [*None*, 5, 10], learning rate: [0.1, 0.01, 0.001] were tuned using grid search.

#### 2.5.4 Random forest

The Random Forest (RF) is a widely used supervised ensemble learning method that can be used for classification and regression problems (Breiman, 2001). As the name suggests RF comprises multiple classifiers which are traditionally Decision Trees (DT) if not otherwise stated. RF builds different classifiers and uses Bagging and feature randomness to reduce the prediction variance and make the model more robust and stable. RF also provides feature importance which can be used for feature selection for downstream tasks. RF reduces the correlation of the individual DT used for the construction of RF by incorporating a random split of the features; however, due to the inclusion of multiple DT, RF is complex and not easily interpretable. The 5-fold cross-validation was used during training, hyper-parameters including the number of estimators [50, 100], maximum depth [*None*, 5, 10], minimum samples required to split a node [2, 5], and minimum samples required at leaf node [1, 4] were tuned using grid search.

#### 2.5.5 Multi-nomial logistic regression

The Logistic Regression (LR) algorithm is a commonly used statistical model for binary classification (McCullagh, 2019). The extension of LR employed for dealing with the case of multiple classes is called Multi-nomial Logistic Regression (MLR). Let *X* represent the features for the independent variables, and *Y* be the true labels. *Y* has labels for more than two distinct classes. In the present case, there will be five distinct labels in *Y* and $X_{i}\in R^{1\times 77}$. For every five classes, we can learn a separate set of weights *W*_*k*_. Let us define the discriminant function in Equation 11, and to calculate the probabilities for each class we need to use the softmax function.


(11) $$\begin{array}{l} f\left(W_{k},X\right)=W_{k}^{⊺}\times X \end{array}$$

(12) $$\text{P}(X_{i}\in k))=\frac{e^{f(W_{k},X_{i})}}{1+\sum_{k=1}^{K-1}e^{f(W_{k},X_{i})}}$$

The Equation 12 gives the probability that the sample unit *i* with features *X*_*i*_ belongs to a specific class. The 5-fold cross-validation was used, and the hyper-parameters for the regularization as *L*1 and *L*2 were searched using grid search. The values considered for the regularization are [0.01, 0.1, 1, 10, 20]. A Saga solver was used to determine the optimal estimates for the model parameters.

The sklearn package in Python was utilized to implement all machine learning models (Pedregosa et al., 2011).

### 2.6 Deep learning classifiers

#### 2.6.1 Baseline model

A baseline model with 5 fully connected dense hidden layers was developed. A batch normalization layer and a ReLU activation follow each linear layer. The layers have 1, 024, 512, 128, 64, and 5 neurons, respectively.

#### 2.6.2 LSTM

Proposed by Hochreiter and Schmidhuber (1997) Long Short Term Memory (LSTM), solves the vanishing gradient problem of Recurrent Neural Networks (RNN). LSTMs find their application in sequence modeling tasks (Sundermeyer et al., 2012; Cao et al., 2019; Yu et al., 2019; Zhang et al., 2020). These sequence modeling applications may include DNA analysis, speech recognition, time series prediction, etc. Using a gating mechanism LSTM can capture the long-term dependencies in the data by maintaining cell states. LSTM uses three gates called output, forget, and update gates to learn the temporal dependence in the data. Let *x*_*t*_ ∈ *R*^*d*^ be the multivariate time series input, *a*_*t*_ be the activation, γ_*t*_ be the cell state at the instance *t*. Corresponding to these *W*_γ_ = [*W*_γ*a*_:*W*_γ*x*_], *W*_*u*_ = [*W*_*ua*_:*W*_*ux*_], *W*_*f*_ = [*W*_*fa*_:*W*_*fx*_], *W*_*o*_ = [*W*_*oa*_:*W*_*ox*_], *b*_γ_, *b*_*u*_, *b*_*f*_, and *b*_*o*_ are the learnable parameters (weights and biases) associated with the cell state, forget, output and update gates. θ_*f*_, θ_*u*_, θ_*o*_ represents the output for the forget, update, and output gates. Non-linear activation functions *tanh* and σ are used in LSTM, the computations for the forward pass through LSTM can be stated as follows


(13) $$\gamma^{'t}=tanh\left(W_{\gamma a}.a_{t-1}+W_{\gamma x}.x_{t}+b_{\gamma }\right)$$

(14) $$\begin{array}{l} \theta_{u}=\sigma \left(W_{ua}.a_{t-1}+W_{ux}.x_{t}+b_{u}\right) \end{array}$$

(15) $$\begin{array}{l} \theta_{f}=\sigma \left(W_{fa}.a_{t-1}+W_{fx}.x_{t}+b_{f}\right) \end{array}$$

(16) $$\begin{array}{l} \theta_{o}=\sigma \left(W_{oa}.a_{t-1}+W_{ox}.x_{t}+b_{o}\right) \end{array}$$

(17) $$\gamma_{t}=\sigma \left(\theta_{u}*\gamma_{t}^{'}+\theta_{f}*\gamma_{t-1}\right)$$

(18) $$\begin{array}{l} a_{t}=\sigma \left(\theta_{0}*\gamma_{t}\right) \end{array}$$

The candidate new cell state is given by $\gamma_{t}^{'}$, (.) is the dot product and (*) is the hadamard product (element wise). The cell state at instance *t* in Equation 17 is expressed as a linear combination of the candidate cell state at instance *t* Equation 13 and cell state at instance *t*−1. These states are scaled by the output of update θ_*u*_ and forget θ_*f*_ gates. In a multi-class classification problem, LSTM units are followed by a Dense layer with a softmax activation which computes the class conditional probabilities to minimize the cross-entropy loss. We used a 5 layer-deep network for multi-class classification. The first 2 layers are LSTM layers with 50 units, followed by 2 dense layers with 50 and 20 units each with Relu activation. Each of these layers is regularized using L2 regularization and followed by batch normalization layers. The output layer has 5 units with a softmax activation.

#### 2.6.3 Bi-directional LSTM

Standard LSTM/RNN blocks are based on the recurrence of past information to the future time steps. However, in many sequence modeling tasks like named entity recognition, etc., to infer the information about the current time step, informatBatch normalization layers follow these layers in a time series context; these models depend on future information as well. Given a sequence of time series, we can use bi-directional LSTM where the recurrence is calculated based on hidden states and gating mechanisms from the past and future (Huang et al., 2015). We constructed a bi-directional LSTM for multi-class classification of fNIRS finger-tapping time-series event averages data. The deep learning model consists of 5 layers, 3 of which are bi-directional LSTM layers with 50, 50, and 20 units. These layers are followed by batch normalization layers. Stacked bi-directional LSTM layers are followed by a dense layer with 20 units with Relu activation. Stacked bi-directional LSTM layers followed by a dense layer are regularized using L2 regularization. The output layer consists of 5 units with softmax activation.

#### 2.6.4 LSTM-CNN hybrid

LSTM-CNN hybrid architectures have shown promising results in time series classification tasks Liu et al. (2019a,b); Garcia et al. (2020); Xie et al. (2020). We use a model with 2 separate heads to process the time series data using 1D convolution and Bi-directional LSTM layers. The CNN head consists of 3 stacked 1D-convolution layers with 64, 64, and 32 filters followed by a dense layer with 32 units. These layers have kernel sizes of 3, 5, and 3 with the same padding, L2-kernel regularization, and Leaky Relu activation. All these layers are followed by batch-normalization layers. LSTM head consists of 3 stacked LSTM layers with 64, 64, and 32 units followed by a dense layer with 32 units with a Leaky Relu activation. All layers are regularized using L2 regularization and followed by batch-normalization layers. The output from LSTM and CNN heads is concatenated and passed to a dense block which has 2 layers with 32 units followed by batch normalization layers with Leaky Relu activation. The output of the dense block is passed to the output layer with 5 units and softmax activation.

#### 2.6.5 Hemo-Net

**Figure 4:**
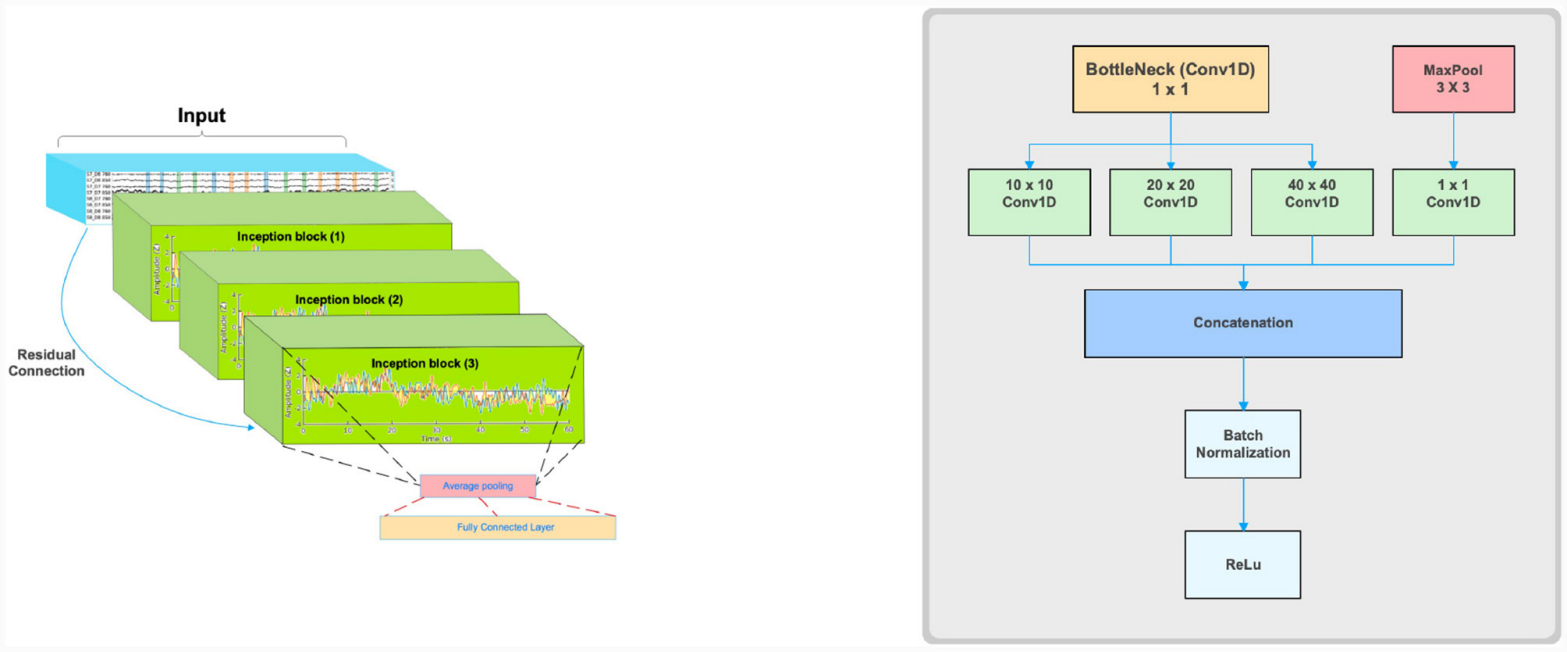
Schematic illustrations of Inception time model adaptation for Hemo-Net **(left)** and a single Inception block **(right)**.

We propose a modified model for multiclass classification of fNIRS data in particular tasks like finger tapping, inspired from Ismail Fawaz et al. (2020). The model is named Hemo-Net after the inspiration from both hemodynamic response and neural network. This model is based on the inception time model and has three inception blocks. The model is shown in Figure 4, which is composed of three inception blocks followed by average pooling and a classifier layer. Our model uses the same inception blocks described in the (Ismail Fawaz et al., 2020). The details of the inception block are given in Figure 4. The inception block comprises bottleneck, max pooling, convolution, and batch-normalization layers followed by a ReLU activation. The main advantage of using the Inception block is that it allows convolving the same input with varying kernel sizes. In this work, we use the kernels of dimensions 10 × 10, 20 × 20, and 40 × 40 based on experimentation inception blocks followed by average pooling layers to help reduce the dimensionality of the data. The pooling layer is connected to a fully connected classification layer with softmax activation. Residual connection is used in the model to avoid the problem of vanishing gradients.

## 3 Results and discussion

**Figure 5:**
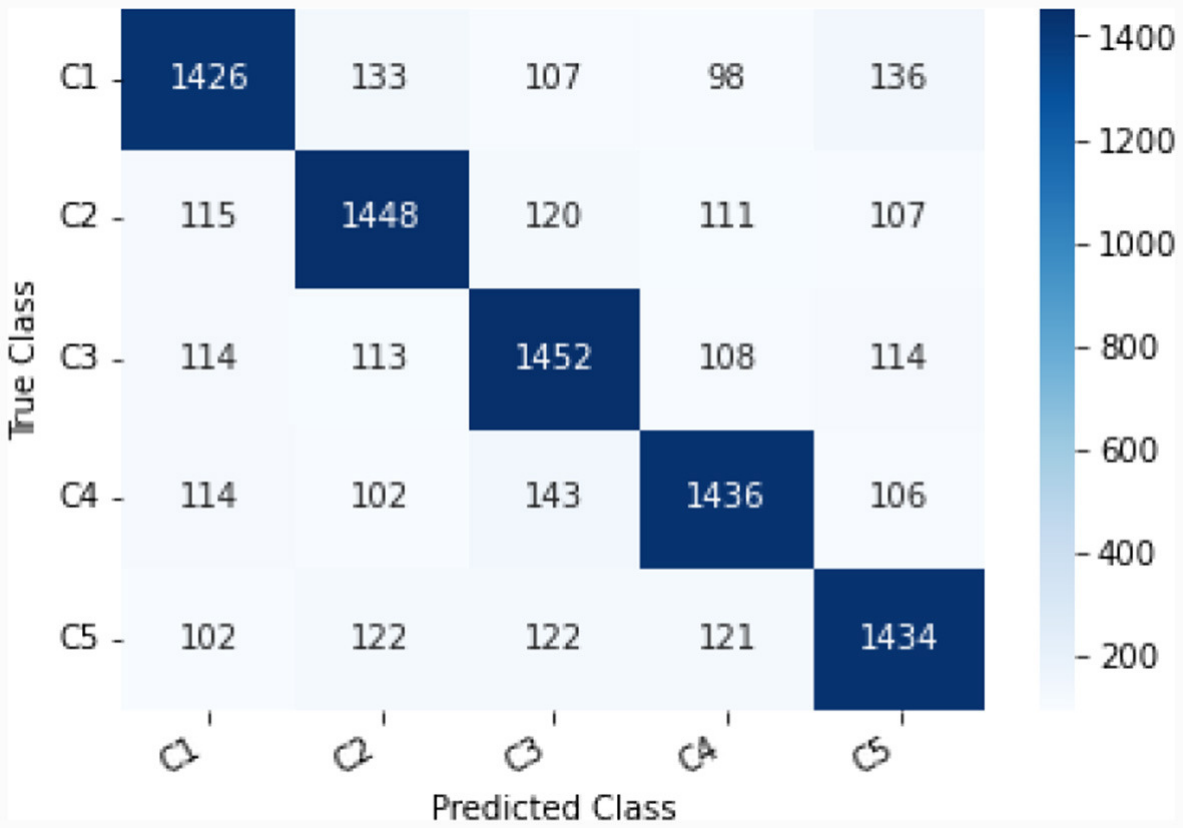
Confusion matrix C1-C5 represent respective class as shown in Table 1.

This study explores the potential of robust information about the subtle motor movements in fNIRS signals with classical ML and DL techniques. We evaluated the performance of various machine learning and deep learning algorithms on the multi-class classification task. We considered 5 machine learning models, including MNLR, QDA, LDA, RF, XGBoost, and 5 deep learning models DNN, LSTM, Bi-directional LSTM, LSTM-CNN Hybrid, and Hemo-Net. The Hemo-Net model is inspired by Ismail Fawaz et al. (2020) which consists of inception blocks that allow learning filters with different dimensions in a single block to extract the useful features from the time series by back-propagation. Among all the models (including machine learning and deep learning), the DL-based model Haemo Net has shown superior performance on the test set. Among machine learning models RF has depicted a superior test set performance. Haemo-Net depicted a test accuracy score of 76%. The confusion matrix is given in Figure 5. The precision is around 75% for each class. We have not observed a high variance between the precision of each class on the test set for Haemo-Net. One reason for this balance can be the balance in the data set for all of the classes, in our training data, we have the same number of examples for the model to learn for all five classes. Recall is also balanced for all of the five classes around 75%. The F1 score which is the harmonic mean of precision and recall and is generally used for the evaluation of the multi-class classifiers is also 76%, which means that all of the classes are being predicted with good precision and recall, the classification report of the model is given in Table 1. We compared the performance of different machine learning and deep learning classifiers. The performance of all the machine and deep learning models on the training and test data is listed in Tables 2, 3 respectively. RF, XGBoost, and Hemo-Net depicted overfitting, while LDA, QDA, and MNLR did not depict this phenomenon. The reason for this behavior can be attributed to the complexity of the models. RF, XGBoost, and Hemo-Net are comparatively complex algorithms involving larger learnable parameters when compared to LDA, QDA, and MNLR. The simple models like LDA, QDA, and MNLR could not perform well on either training or test data. The QDA algorithm achieved a better performance in terms of accuracy on the test set with a score of 51.3%. Among the overfitting models, DL-based Hemo-Net performs better on the test set than the LSTM, DNN, LSTM-CNN hybrid ensemble-based methods of RF and XGBoost.

**Table 1:** Hemo-Net classification report test data.

**Finger (Class label)**	**Precision**	**Recall**	**F1-Score**	**Support**
Thumb (C1)	0.76	0.75	0.76	1,900
Index (C2)	0.75	0.76	0.76	1,901
Middle (C3)	0.75	0.76	0.76	1,901
Ring (C4)	0.77	0.76	0.76	1,901
Little (C5)	0.76	0.75	0.76	1,901
Accuracy			0.76	9,504
Macro Avg.	0.76	0.76	0.76	9,504
Weighted Avg.	0.76	0.76	0.76	9,504

**Table 2:** Machine learning models performance.

**Model**	**Training accuracy (%)**	**Test accuracy (%)**	**HyperParameters**
Random forest (RF)	100	60.2	Max depth: None, Min samples leaf: 1, Min samples split: 2, N estimators: 100
XGBoost	95.9	57.4	Max depth: 10, Learning rate: 0.1, N estimators: 100
Linear discriminant analysis (LDA)	33.4	31.9	shrinkage: None, solver: lsqr
Quadratic discriminant analysis (QDA)	54.9	51.3	reg param: 0
Multinomial logistic regression (MNLR)	30.8	29.7	C : 20, penalty: L2

**Table 3:** Deep learning models performance.

**Model**	**Training accuracy (%)**	**Test accuracy (%)**	**HyperParameters**
Baseline DL model	96	68	Batch size: 32, penalty: L2, Learning rate: Cyclic [0.01–0.00001]
Hemo-Net(Ours)	96	76	Batch size: 32, penalty: L2, Learning rate: Cyclic [0.01–0.00001]
LSTM	74	56	Batch size: 32, penalty: L2, Learning rate: 1e-4
Bi-directional LSTM	96	63	Batch size: 32, penalty: L2, Learning rate: 1e-4
CNN-LSTM Hybrid	94	63	Batch size: 32, penalty: L2, Learning rate: 1e-4

The results demonstrate the potential rich information in the fNIRS signal to represent and classify fine anatomical movements such as decoding individual finger tapping. The classifier has been demonstrated to perform remarkably better than the baseline models. This model can serve as a baseline for training more complex models using big data because DL models tend to perform better when trained on large data sets, as observed previously. Also, our model can be used for transfer learning for similar tasks like foot tapping, etc. With pre-trained weights, we can further improve the accuracy of our model which can be used to classify various related fNIRS-based BCI tasks. The classification accuracy achieved with our Hemo-Net model for five class problems was also much approved compared to our previous work (Khan et al., 2021b) for binary classification of the same data with classical ML models which was approximately (77% for XGBoost). The multi-class classification problem is comparatively complex to learn for machine learning models compared to the binary classification task. The brain imaging data sets are generally based on the design of experiment strategies and are therefore limited in the size of the data. By conducting more experiments and collecting more data for the finger-tapping task the accuracy of the model can be improved. One possible direction with limited data sets can be to explore deep generative models for data augmentation and then retrain the classifier with the augmented data.

There are different sources of randomness that might be contributing to the 25% error in classification. There is variation in the person-to-person tapping pattern and corresponding brain activation. There is also variation within the trials or replication of the same person. The future model can include these random effects to make more robust classifiers. We also identify that event averages might also be deteriorating the inherited rich information in the raw signals, therefore in future works, we can experiment with models to extract the features directly from the raw or minimally processed data. This can also be helpful in real-time applications as with minimal pre-processing of the data and taking total leverage of the data-driven DL technologies, the lags in the BCI can be reduced.

Our primary focus in this study is to investigate the classification of fine anatomical movements, specifically individual finger-tapping tasks, utilizing fNIRS. By exploring the potential of fNIRS in brain-computer interface (BCI) applications, we aim to enhance our understanding of the hemodynamic responses associated with such movements. In the future, our research will expand to include the localization and temporal response analysis of brain activity by utilizing hybrid EEG-fNIRS techniques. In the future, if we can accurately measure finger movements using fNIRS and classify the signals correctly, it could have several advantages from a biomedical perspective. These include improved prosthetic control, enhanced rehabilitation assessment and monitoring, better understanding and treatment of neurological disorders, advancements in human-computer interaction, development of brain-machine interfaces, and the potential for cognitive assessment. This technology has the potential to significantly benefit individuals with motor impairments and neurological conditions, providing them with greater control, functionality, and improved quality of life. Our ultimate goal is to develop effective and efficient BCI systems that can improve the quality of life for individuals with motor impairments.

## 4 Limitations and future work

In this work, we analyzed the performance of different ML and DL algorithms on the task of multi-class classification of finger tapping from the event averages data. We observed that the DL-based architecture Hemo-Net has a better performance, but suffers from overfitting. This can be attributed to the data scarcity and the model's complexity. We propose to extend our work to collect more data for these complex models while utilizing transfer learning to initialize the models from the pre-learned weights on this data set. Event averages have been currently used for the fNIRS channels however event averages can destroy the temporal dependence structure in the data. Therefore we can build the models on the raw time series instead of event averages and compare the difference in the evaluation metrics for the classifiers for both approaches. Moreover using raw signals with minimal pre-processing also is beneficial for the real-time application of these classifiers on embedded systems.

Generative AI and DL methods can also be explored for building robust classifiers. The data can be augmented by using Generative Adversarial Neural Networks (GANs) or Variational Auto Encoders (VAEs) for the 5 classes. The classifiers can then be trained on these augmented data, and the real data can be used for evaluation in 3-folds with training, testing, and validation, with the test set never used for augmentation. The hypothesis behind this approach is that each generative model for the classes is assumed to capture the class-specific patterns in the data while minimizing the randomness from the uncontrolled sources (sensing, noise, inter-person tapping variation, intra-person tapping variation).

## 5 Conclusion

We explore the potential of functional near-infrared spectroscopy (fNIRS) in classifying fine anatomical movements using classical, modern machine learning (ML), and deep learning (DL) approaches. The results are promising, demonstrating acceptable accuracies for such challenging tasks that involve classifying fine anatomical movements. These findings highlight the potential of fNIRS signals in capturing information related to fine movements. However, to further enhance the application of brain-computer interfaces (BCIs), we require larger datasets, improved models, and reduced computational requirements. The proposed model, Hemo-Net exhibits superior performance compared to others. It has been observed that complex models tend to overfit the training data but perform better when evaluated on the test set than simpler models. This suggests the complexity of the problem at hand and the limited size of the available data. Collecting more future data can help improve these models' performance. Instead of training Hemo-Net from scratch, we propose training it with additional data using the pre-learned weights obtained from this study. This approach may decrease the training time required for the model.

## Data availability statement

The raw data supporting the conclusions of this article will be made available by the authors, without undue reservation.

## Ethics statement

The studies involving humans were approved by Norwegian Centre for Research Data. The studies were conducted in accordance with the local legislation and institutional requirements. The participants provided their written informed consent to participate in this study.

## Author contributions

HK: Conceptualization, Formal analysis, Investigation, Validation, Writing – original draft, Writing – review & editing. RK: Formal analysis, Methodology, Software, Visualization, Writing – review & editing. MS: Formal analysis, Methodology, Software, Visualization, Writing – original draft, Writing – review & editing. AY: Supervision, Validation, Writing – review & editing. HO: Supervision, Validation, Writing – review & editing. PM: Project administration, Supervision, Validation, Writing – review & editing.

## References

[B1] Ahn S. Jun S. C. (2017). Multi-modal integration of eeg-fnirs for brain-computer interfaces-current limitations and future directions. Front. Hum. Neurosci. 11:503. 10.3389/fnhum.2017.00503 29093673 PMC5651279

[B2] Boas D. A. Strangman G. E. Culver J. P. Hoge R. D. Jasdzewski G. Poldrack R. A.. (2003). Can the cerebral metabolic rate of oxygen be estimated with near-infrared spectroscopy? Phys. Med. Biol. 48, 2405–2418. 10.1088/0031-9155/48/15/311 12953906

[B3] Breiman L. (2001). Random forests. Mach. Learn. 45, 5–32. 10.1023/A:1010933404324

[B4] Cao J. Li Z. Li J. (2019). Financial time series forecasting model based on CEEMDAN and LSTM. Physica Stat. Mech. Applic. 519, 127–139. 10.1016/j.physa.2018.11.061

[B5] Chen T. Guestrin C. (2016). “Xgboost: a scalable tree boosting system,” in Proceedings of the 22nd ACM Sigkdd International Conference on Knowledge Discovery and Data Mining, 785–794. 10.1145/2939672.2939785

[B6] Ferrari M. Quaresima V. (2012). A brief review on the history of human functional near-infrared spectroscopy (FNIRS) development and fields of application. Neuroimage 63, 921–935. 10.1016/j.neuroimage.2012.03.049 22510258

[B7] Fishburn F. A. Ludlum R. S. Vaidya C. J. Medvedev A. V. (2019). Temporal derivative distribution repair (TDDR): a motion correction method for fnirs. Neuroimage 184, 171–179. 10.1016/j.neuroimage.2018.09.025 30217544 PMC6230489

[B8] Fisher R. A. (1936). The use of multiple measurements in taxonomic problems. Ann. Eugen. 7, 179–188. 10.1111/j.1469-1809.1936.tb02137.x

[B9] Gagnon L. Yücel M. A. Dehaes M. Cooper R. J. Perdue K. L. Selb J.. (2012). Quantification of the cortical contribution to the nirs signal over the motor cortex using concurrent nirs-fmri measurements. Neuroimage 59, 3933–3940. 10.1016/j.neuroimage.2011.10.054 22036999 PMC3279595

[B10] Gannouni S. Belwafi K. Aboalsamh H. AlSamhan Z. Alebdi B. Almassad Y.. (2020). EEG-based BCI system to detect fingers movements. Brain Sci. 10:965. 10.3390/brainsci10120965 33321915 PMC7763179

[B11] Garcia C. I. Grasso F. Luchetta A. Piccirilli M. C. Paolucci L. Talluri G. (2020). A comparison of power quality disturbance detection and classification methods using CNN, LSTM and CNN-LSTM. Applied sciences 10, 6755. 10.3390/app10196755

[B12] Hastie T. Tibshirani R. Friedman J. H. Friedman J. H. (2009). The Elements of Statistical Learning: Data Mining, Inference, and Prediction, volume 2. Cham: Springer. 10.1007/978-0-387-84858-7

[B13] Hochreiter S. Schmidhuber J. (1997). Long short-term memory. Neural Comput. 9, 1735–1780. 10.1162/neco.1997.9.8.1735 9377276

[B14] Holper L. Wolf M. (2011). Single-trial classification of motor imagery differing in task complexity: a functional near-infrared spectroscopy study. J. Neuroeng. Rehabilit. 8, 1–13. 10.1186/1743-0003-8-34 21682906 PMC3133548

[B15] Hong K.-S. Khan M. J. (2017). Hybrid brain-computer interface techniques for improved classification accuracy and increased number of commands: a review. Front. Neurorob. 11, 35. 10.3389/fnbot.2017.00035 PMC5522881 28790910

[B16] Hong K. S. Khan M. J. Hong M. J. (2018). Feature extraction and classification methods for hybrid fnirs-eeg brain-computer interfaces. Front. Hum. Neurosci. 12:246. 10.3389/fnhum.2018.00246 30002623 PMC6032997

[B17] Huang Z. Xu W. Yu K. (2015). Bidirectional lstm-crf models for sequence tagging. arXiv preprint arXiv:1508.01991.

[B18] Ismail Fawaz H. Lucas B. Forestier G. Pelletier C. Schmidt D. F. Weber J.. (2020). Inception time: finding a lexnet for time series classification. Data Min. Knowl. Discov. 34, 1936–1962. 10.1007/s10618-020-00710-y

[B19] Johnson R. A. Wichern D. W.. (2002). Applied Multivariate Statistical Analysis. Upper Saddle River, NJ: Prentice Hall.

[B20] Jorge A. Royston D. A. Tyler-Kabara E. C. Boninger M. L. Collinger J. L. (2020). Classification of individual finger movements using intracortical recordings in human motor cortex. Neurosurgery 87, 630–638. 10.1093/neuros/nyaa026 32140722

[B21] Khan H. Naseer N. Yazidi A. Eide P. K. Hassan H. W. Mirtaheri P. (2021a). Analysis of human gait using hybrid EEG-FNIRS-based BCI system: a review. Front. Hum. Neurosci. 14:613254. 10.3389/fnhum.2020.613254 33568979 PMC7868344

[B22] Khan H. Noori F. M. Yazidi A. Uddin M. Z. Khan M. A. Mirtaheri P. (2021b). Classification of individual finger movements from right hand using FNIRS signals. Sensors 21:7943. 10.3390/s21237943 34883949 PMC8659988

[B23] Khan M. A. Bhutta M. R. Hong K.-S. (2020). Task-specific stimulation duration for FNIRS brain-computer interface. IEEE Access 8, 89093–89105. 10.1109/ACCESS.2020.2993620

[B24] Kilmarx J. Oblak E. Sulzer J. Lewis-Peacock J. (2021). Towards a common template for neural reinforcement of finger individuation. Sci. Rep. 11:1065. 10.1038/s41598-020-80166-8 33441742 PMC7806844

[B25] LaConte S. M. Peltier S. J. Hu X. P. (2007). Real-time fMRI using brain-state classification. Hum. Brain Mapp. 28, 1033–1044. 10.1002/hbm.20326 17133383 PMC6871430

[B26] Liao K. Xiao R. Gonzalez J. Ding L. (2014). Decoding individual finger movements from one hand using human EEG signals. PLoS ONE 9:e85192. 10.1371/journal.pone.0085192 24416360 PMC3885680

[B27] Liu F. Zhou X. Cao J. Wang Z. Wang H. Zhang Y. (2019a). “A LSTM and CNN based assemble neural network framework for arrhythmias classification,” in ICASSP 2019–2019 IEEE International Conference on Acoustics, Speech and Signal Processing (ICASSP) (IEEE), 1303–1307. 10.1109/ICASSP.2019.8682299

[B28] Liu F. Zhou X. Wang T. Cao J. Wang Z. Wang H.. (2019b). “An attention-based hybrid LSTM-CNN model for arrhythmias classification,” in 2019 International Joint Conference on Neural Networks (IJCNN) (IEEE), 1–8. 10.1109/IJCNN.2019.8852037

[B29] Lotte F. Bougrain L. Cichocki A. Clerc M. Congedo M. Rakotomamonjy A.. (2018). A review of classification algorithms for EEG-based brain-computer interfaces: a 10 year update. J. Neural Eng. 15, 031005. 10.1088/1741-2552/aab2f2 29488902

[B30] Lotte F. Congedo M. Lécuyer A. Lamarche F. Arnaldi B. (2007). A review of classification algorithms for EEG-based brain-computer interfaces. J. Neural Eng. 4:R1. 10.1088/1741-2560/4/2/R01 17409472

[B31] McCullagh P. (2019). Generalized Linear Models. London: Routledge. 10.1201/9780203753736

[B32] Middendorf M. McMillan G. Calhoun G. Jones K. S. (2000). Brain-computer interfaces based on the steady-state visual-evoked response. IEEE Trans. Rehabilit. Eng. 8, 211–214. 10.1109/86.847819 10896190

[B33] Naseer N. Hong K.-S. (2015). fnirs-based brain-computer interfaces: a review. Front. Hum. Neurosci. 9:3. 10.3389/fnhum.2015.00003 25674060 PMC4309034

[B34] Nazeer H. Naseer N. Khan R. A. Noori F. M. Qureshi N. K. Khan U. S.. (2020). Enhancing classification accuracy of FNIRS-BCI using features acquired from vector-based phase analysis. J. Neural Eng. 17:056025. 10.1088/1741-2552/abb417 33055382

[B35] Pedregosa F. Varoquaux G. Gramfort A. Michel V. Thirion B. Grisel O.. (2011). Scikit-learn: machine learning in Python. J. Mach. Learn. Res. 12, 2825–2830.

[B36] Pfurtscheller G. Allison B. Z. Bauernfeind G. Brunner C. Solis Escalante T. Scherer R.. (2010). The hybrid BCI. Front. Neurosci. 4:3. 10.3389/fnpro.2010.00003 20582271 PMC2891647

[B37] Sato H. Kiguchi M. Kawaguchi F. Maki A. (2004). Practicality of wavelength selection to improve signal-to-noise ratio in near-infrared spectroscopy. Neuroimage 21, 1554–1562. 10.1016/j.neuroimage.2003.12.017 15050579

[B38] Shen G. Zhang J. Wang M. Lei D. Yang G. Zhang S.. (2014). Decoding the individual finger movements from single-trial functional magnetic resonance imaging recordings of human brain activity. Eur. J. Neurosci. 39, 2071–2082. 10.1111/ejn.12547 24661456

[B39] Shin J. Jeong J. (2014). Multiclass classification of hemodynamic responses for performance improvement of functional near-infrared spectroscopy-based brain-computer interface. J. Biomed. Optics 19:067009. 10.1117/1.JBO.19.6.067009 24967916

[B40] Sitaram R. Caria A. Veit R. Gaber T. Rota G. Kuebler A.. (2007). FMRI brain-computer interface: a tool for neuroscientific research and treatment. Comput. Intell. Neurosci. 2007:25487. 10.1155/2007/25487 18274615 PMC2233807

[B41] Strangman G. Culver J. P. Thompson J. H. Boas D. A. (2002). A quantitative comparison of simultaneous bold fmri and nirs recordings during functional brain activation. Neuroimage 17, 719–731. 10.1006/nimg.2002.1227 12377147

[B42] Strangman G. Franceschini M. A. Boas D. A. (2003). Factors affecting the accuracy of near-infrared spectroscopy concentration calculations for focal changes in oxygenation parameters. Neuroimage 18, 865–879. 10.1016/S1053-8119(03)00021-1 12725763

[B43] Sundermeyer M. Schlüter R. Ney H. (2012). “LSTM neural networks for language modeling,” in Thirteenth annual Conference of the International Speech Communication Association. 10.21437/Interspeech.2012-65

[B44] Warbrick T. (2022). Simultaneous eeg-fmri: what have we learned and what does the future hold? Sensors 22:2262. 10.3390/s22062262 35336434 PMC8952790

[B45] Wilcox T. Biondi M. (2015). fnirs in the developmental sciences. Cogn. Sci. 6, 263–283. 10.1002/wcs.1343 26263229 PMC4979552

[B46] Xie H. Zhang L. Lim C. P. (2020). Evolving cnn-lstm models for time series prediction using enhanced grey wolf optimizer. IEEE access 8, 161519–161541. 10.1109/ACCESS.2020.3021527

[B47] Yao L. Zhu B. Shoaran M. (2022). Fast and accurate decoding of finger movements from ecog through riemannian features and modern machine learning techniques. J. Neural Eng. 19, 016037. 10.1088/1741-2552/ac4ed1 35078156

[B48] Yu Y. Si X. Hu C. Zhang J. (2019). A review of recurrent neural networks: Lstm cells and network architectures. Neural Comput. 31, 1235–1270. 10.1162/neco_a_01199 31113301

[B49] Zanos S. Miller K. J. Ojemann J. G. (2008). “Electrocorticographic spectral changes associated with ipsilateral individual finger and whole hand movement,” in 2008 30th Annual International Conference of the IEEE Engineering in Medicine and Biology Society (IEEE), 5939–5942. 10.1109/IEMBS.2008.4650569 19164072

[B50] Zeiler M. D. Fergus R. (2014). “Visualizing and understanding convolutional networks,” in European Conference on Computer Vision (Springer), 818–833. 10.1007/978-3-319-10590-1_53

[B51] Zhang Y. Qiao S. Ji S. Li Y. (2020). Deepsite: bidirectional lstm and cnn models for predicting dna-protein binding. Int. J. Mach. Learn. Cyber. 11, 841–851. 10.1007/s13042-019-00990-x

